# Evaluation of the Golden Proportion, Golden Percentage, and Recurring Esthetic Dental Proportion in Kenyans of African Descent

**DOI:** 10.1002/cre2.923

**Published:** 2024-07-05

**Authors:** Marion N. Mosomi, Susan W. Maina, Olivia A. Osiro, Ben I. Omondi

**Affiliations:** ^1^ Department of Dental Sciences University of Nairobi Dental Hospital Nairobi Kenya

**Keywords:** Golden Percentage, Golden Proportion, maxillary anterior tooth proportions, Recurring Esthetic Dental Proportion

## Abstract

**Objectives:**

To evaluate the validity of the Golden Proportion, Golden Percentage, and Recurring Esthetic Dental (RED) Proportion among Kenyans of African descent with naturally well‐aligned teeth.

**Materials and Methods:**

Standardized frontal photographic images of the smiles of 175 participants aged 18–35 years were obtained, and Adobe Photoshop was used to analyze and measure the frontal widths of the maxillary central and lateral incisors and canines in triplicate. The average teeth widths were calculated to determine the existence of the Golden Proportion, Golden Percentage, and RED Proportion, and their validity using independent sample *t*‐tests to compare the differences in the mean teeth widths at *α* < 0.05.

**Results:**

The number of male and female participants was 107 (61.1%) and 68 (38.9%), respectively. The Golden Proportion between the maxillary central and lateral incisors was found in 4.0% on the right and 2.8% on the left of all the participants, but between the maxillary lateral incisors and canines was found in only 0.6% on the right of male participants (*p* < 0.0001). The RED Proportion between the maxillary lateral and central incisors was in the range of 67%–70%, and between the canines and lateral incisors was 82%–84% (*p* < 0.0001). The proportion of RED was not constant, and gradually increased distally. The Golden Percentage of 15% was observed in the lateral incisors bilaterally; however, in the central incisors and the canines, the Golden Percentage was 22% and 12%, respectively.

**Conclusion:**

The Golden and RED Proportions were invalid determinants of anterior teeth proportions. The Golden Percentage existed only in the lateral incisors. The Golden Proportion, RED Proportion, and Golden Percentage theories may not be applicable to all populations when designing smiles. Racial and ethnic backgrounds are important considerations to establish objective quantifiable values of anterior tooth proportions that are beneficial for esthetic restorations.

## 1 Introduction

Dental esthetics are a major contributor to the oral health‐seeking behavior of patients globally. This may be driven by dental diseases such as dental caries, discoloration arising from conditions such as dental fluorosis, developmental dental defects such as amelogenesis and dentinogenesis imperfecta, malocclusion, and oral and maxillofacial trauma. In addition to their unsightly appearance, these conditions may lead to tooth loss or edentulism, necessitating treatment with fixed or removable dental prostheses. Esthetic dental treatment and oral rehabilitation are major components of contemporary dental practice globally and are exceedingly costly in developing countries (Bernabé, Masood, and Vujicic 2017).

The proportion of teeth is a major concern for dentists and dental technologists when considering the esthetic aspects of dentistry. Over the years, facial esthetics have been reported to be most pleasant when the width of the central to lateral incisors and lateral incisors to canines have a repeated proportion when viewed from the front (Dalaie et al. 2017). Facial esthetics are also enhanced when the maxillary anterior teeth are dominant in the smile arch (Wagh, Mantri, and Bhasin 2020). A study reported that several factors affect the symmetry, dominance, and proportion of maxillary anterior teeth (Snow 1999); therefore, restorative dentists must consider the patient's subjective concerns when designing a natural smile, along with the objective criteria (Wagh, Mantri, and Bhasin 2020). Some theories have been proposed to assess the size and form of maxillary anterior teeth to ensure symmetry during restorations and replacements, including the Golden Proportion, Golden Percentage, and Recurring Esthetic Dental (RED) Proportion (Mahshid et al. 2004; Mancuso 2004).

The Golden Proportion is a mathematically constant ratio proposed by the Greek mathematician Euclid, which defines the ideal proportions between larger and smaller lengths desirable for an esthetic appearance as 1.618:1.0 (Azimi et al. 2017). The golden ratio is a valuable tool for evaluating symmetry and dominance and is known as the standard for perfect beauty (Azimi et al. 2017; Aziz and Hossain 2017). In 1973, Lombardi was the first to suggest the application of the Golden Proportion in dentistry, proposing the existence of naturally proportionate teeth, but rejecting the idea of using the Golden Proportion to create esthetic teeth. He noted that a recurring ratio existed between all teeth, from the central incisor to the first premolar (Lombardi 1973). Levin observed that the width of the maxillary central incisor was in Golden Proportion to the width of the lateral incisor, as was the width of the lateral incisor to the width of the canine (Levin 1978). He described the visible width of the lateral incisor as 62% (0.618) of the central incisor and that of the canine as 62% (0.618) of the lateral incisor (Levin 2011). Based on this observation, a tooth caliper and Golden Proportion diagnostic grid were developed to perfect the esthetics of the anterior teeth (Rosenstiel, Land, and Walter 2022). The Golden Proportion has been widely accepted as an esthetic guideline for the restoration of anterior teeth (Rosenstiel, Land, and Walter 2022; Rufenacht 1990; Shillingburg et al. 1997; Goldstein et al. 2018).

Nonetheless, others (Snow 1999; Ward 2001; Preston 1993) contradict the validity of the Golden Proportion theory, proposing alternatives such as the Golden Percentage (Snow 1999), Preston's Proportion (Preston 1993), and RED Proportion (Ward 2001), respectively. It was proposed that individual tooth width can be determined as a fixed proportion of each tooth's frontal view being a percentage of the visible inter‐canine width; this was named the Golden Percentage and described as 10% each for left and right canines, 15% each for left and right lateral incisors, and 25% each for left and right central incisors (Snow 1999).

Further, the RED Proportion was introduced after studies on the Golden Proportion found that the lateral incisor appeared narrow, and the canine was not prominent enough (Ward 2001). Clinicians were advised to use a proportion of their choice, as long as it remained consistent when proceeding distally from the midline. The RED Proportion is a concept of proportional smile design based on a linear coefficient progression in which the width of each successive tooth, as viewed from the front, diminishes by the same proportion. It is a measure of the average width of the maxillary lateral incisor in relation to the central incisor and that of the maxillary canine in relation to the lateral incisor, with acceptable values between 60% and 80% (Azimi et al. 2017). Several studies have evaluated the existence and validity of these parameters, with varying and inconclusive results.

One study among Romani men and women reported that the Golden Proportion between the lateral and central incisor occurs in a higher proportion than that between the canine and the lateral incisor (Markovics, Jánosi, and Biriș 2013). Another study among Malaysian dental students reported that fewer than 20% of the subjects had their right lateral incisors and canines in Golden Proportion with the width of their right central incisors and right lateral incisors, respectively (Sulaiman et al. 2010) similar to Iranian dental students among whom the Golden Proportion was not observed between the widths of the canines and that of the lateral incisors (Snow 1999; Mahshid et al. 2004). Others have also reported that the Golden Proportion did not occur in their study subjects (Mahshid et al. 2004; Levin 1978; Goldstein et al. 2018; Sandeep et al. 2015; Fayyad, Jamani, and Aqrabawi 2006; Pesson et al. 2015), suggesting that its existence is uncommon (Snow 1999; Mahshid et al. 2004; Sandeep et al. 2015; Fayyad, Jamani, and Aqrabawi 2006; Pesson et al. 2015; Ramani and Sreenivasan Murthy 2008; Pini et al. 2012; Ward 2007) and may not be essential for the perceived attractiveness of the smile. Regarding the Golden Percentage, two studies reported that their study subjects were in conformity (Ramani and Sreenivasan Murthy 2008; Qadri, 2016); several other studies did not find this to be so (Preston 1993; Markovics, Jánosi, and Biriș 2013; Fayyad, Jamani, and Aqrabawi 2006), but, however, concluded that with minor modifications, the Golden Percentage is more applicable than the Golden Proportion to relate the successive widths of the maxillary anterior teeth if the percentages are adjusted considering the ethnicity of the population. Evaluation of the RED Proportion also revealed values that are not constant, increasing distally from the midline (Fayyad, Jamani, and Aqrabawi 2006; Ramani and Sreenivasan Murthy 2008; Pitti et al. 2011). Most studies evaluating the existence and suitability of maxillary anterior tooth proportions have been performed in European and Asian populations with inconclusive or even contradictory results (Snow 1999; Mahshid et al. 2004; Ward 2001; Ramani and Sreenivasan Murthy 2008; Hasanreisoglu et al. 2005; Shirinzad and Ahmady 2006; Gillen et al. 1994). A study in Kenya concluded that the inter canthal width was unreliable for estimating mesiodistal widths of the maxillary anterior teeth (Ariemba, Gathece, and Maina 2017).

There is scant information on these theories assessing the size and form of the maxillary anterior teeth as determinants of dental esthetics in the African population. Therefore, this study aimed to evaluate the existence of the Golden Proportion, RED Proportion, and Golden Percentage and determine their validity among Kenyans of African descent with naturally well‐aligned teeth. The hypothesis under investigation was that The Golden Proportion, RED Proportion, and Golden Percentage do not exist and are invalid determinants of anterior teeth proportions among Kenyans of African descent with naturally well‐aligned teeth.

## 2 Materials and Methods

### 2.1 Study Site

This study was conducted in January 2022 at three campuses of the Faculty of Health Sciences, University of Nairobi, Nairobi, Kenya: the Department of Dental Sciences which houses the University of Nairobi Dental Hospital, the preclinical departments at Chiromo, and the clinical medical, pharmacy, and nursing departments at Kenyatta National Hospital.

### 2.2 Study Population

The study population comprised students from the three campuses and patients attending the University Dental Hospital. At the time of the study, 66 undergraduate and 25 postgraduate dental students were attending clinical sessions, with an average of 1000 patients in four clinical units. There were 620 medical, 410 pharmacy, and 219 nursing students from the other two campuses. Thus, the estimated study population was 2340 persons.

### 2.3 Inclusion and Exclusion Criteria

The inclusion criteria were as follows: patients aged 18–35 years who consented to participate in the study; undergraduate and postgraduate students who consented to participate in the study; patients and students with natural dentition and well‐aligned teeth, as defined by Andrew's Six Keys of occlusion (Andrews 1972); patients and students with no missing maxillary anterior teeth; and patients and students of Kenyan origin and African descent. The exclusion criteria were as follows: individuals aged <18 and >35 years; with malformed anterior teeth; with crowding of the maxillary anterior teeth; with restorations on the maxillary anterior teeth; with loss of tooth structure due to fracture, attrition, and caries on the maxillary anterior teeth; missing any maxillary anterior teeth; with a history of orthodontic treatment; and non‐Kenyans and Kenyans not of African descent.

### 2.4 Sample Size, Technique, and Recruitment Process

**Figure 1:**
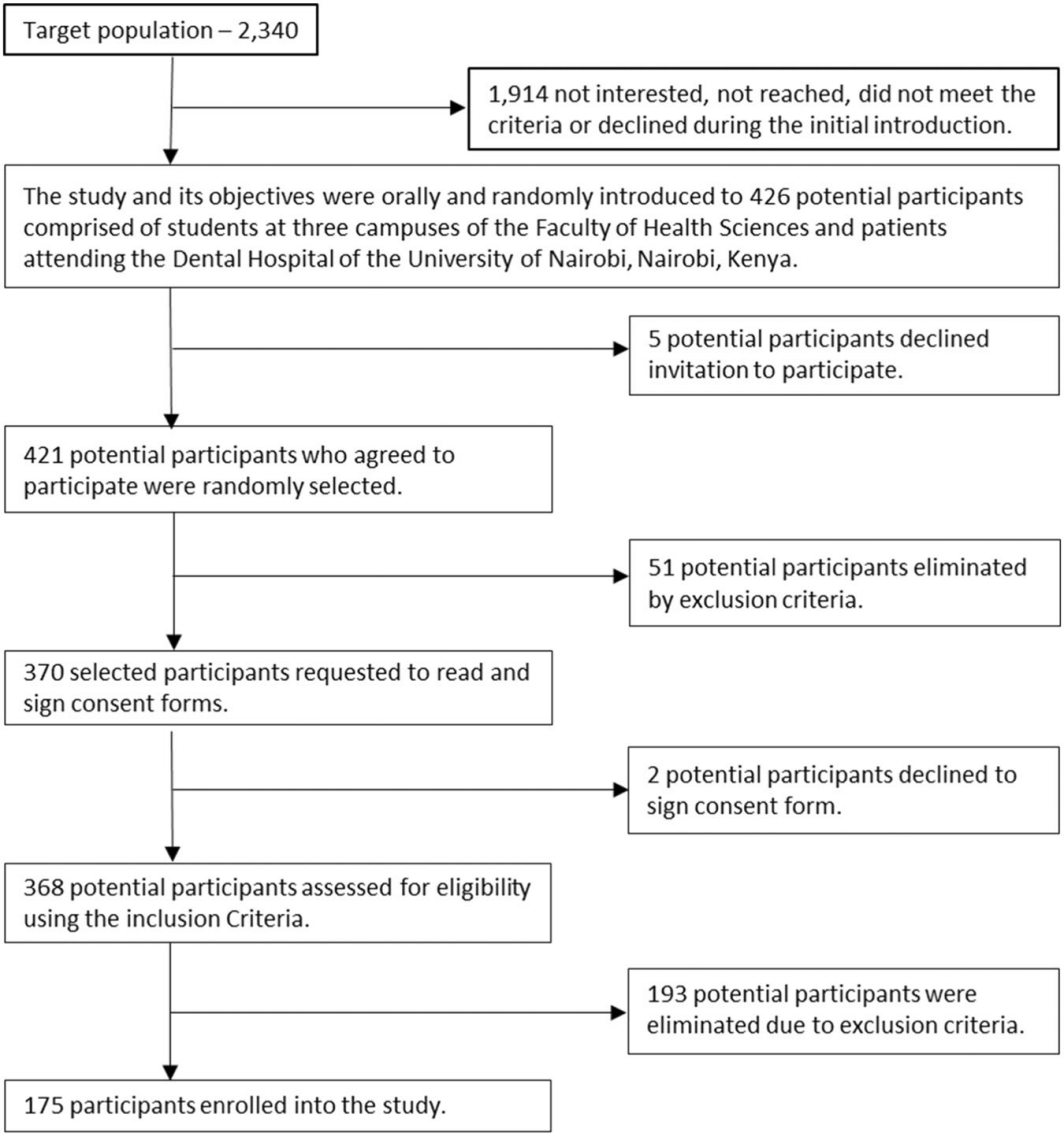
Study participants recruitment process.

Fisher's formula for cross‐sectional studies was used to calculate a minimum sample size of 174 participants, who were recruited as summarized in Figure 1. Ethical approval was obtained from the Kenyatta National Hospital/University of Nairobi Ethics and Research Committee (KNH‐ERC/A/173). Briefly, research assistants randomly approached groups of students in lecture theatres at the three campuses and patients in the waiting rooms of various clinics at the University Dental Hospital. They were briefed orally about the study and its objectives, thereby randomly identifying those who were interested. Afterward, potential participants were approached individually by the research assistants and requested to read and sign consent forms during the same session or to carry the consent form home and consult with a study counselor of their choice, if necessary, before consenting to participate in the study. Potential participants who completed the consent process were interviewed, screened, and recruited if they met the inclusion criteria. The examination of the oral cavity was performed by the first author M.N.M. in the conservation clinic at the University Dental Hospital and lecture theatres at other study sites.

### 2.5 Data Collection Tools and Procedure

After recruitment, participants were briefed again on the study and its objectives. Following confirmation of consent, they were subjected to an oral interview to gather information on their sociodemographic characteristics. For confidentiality purposes, the names and contacts of the participants were not recorded, and the data sheets were serialized. The participants were asked to display their widest smile to reveal all the maxillary anterior teeth to confirm that they were naturally well aligned. Those who consented to continue in the study were then invited to the conservation clinic at the University Dental Hospital or lecture theatres in the three campuses for a final screening to ensure that they met the inclusion criteria and were prepared to have their photographs taken.

### 2.6 Photography Procedure

Photographs were taken under natural lighting between 10 am and 12 noon with the participants seated in an upright position on an ordinary chair. All photographs of the participants’ smiles were captured by the first author M.N.M. Photographs were taken at a set distance of 1.5 m to ensure a safe physical distance between the participant and M.N.M. Furthermore, to ensure adequate time for the disinfection of the examination chair between participants, M.N.M. aimed to examine and take photographs of a maximum of six participants per session in the morning.

**Figure 2:**
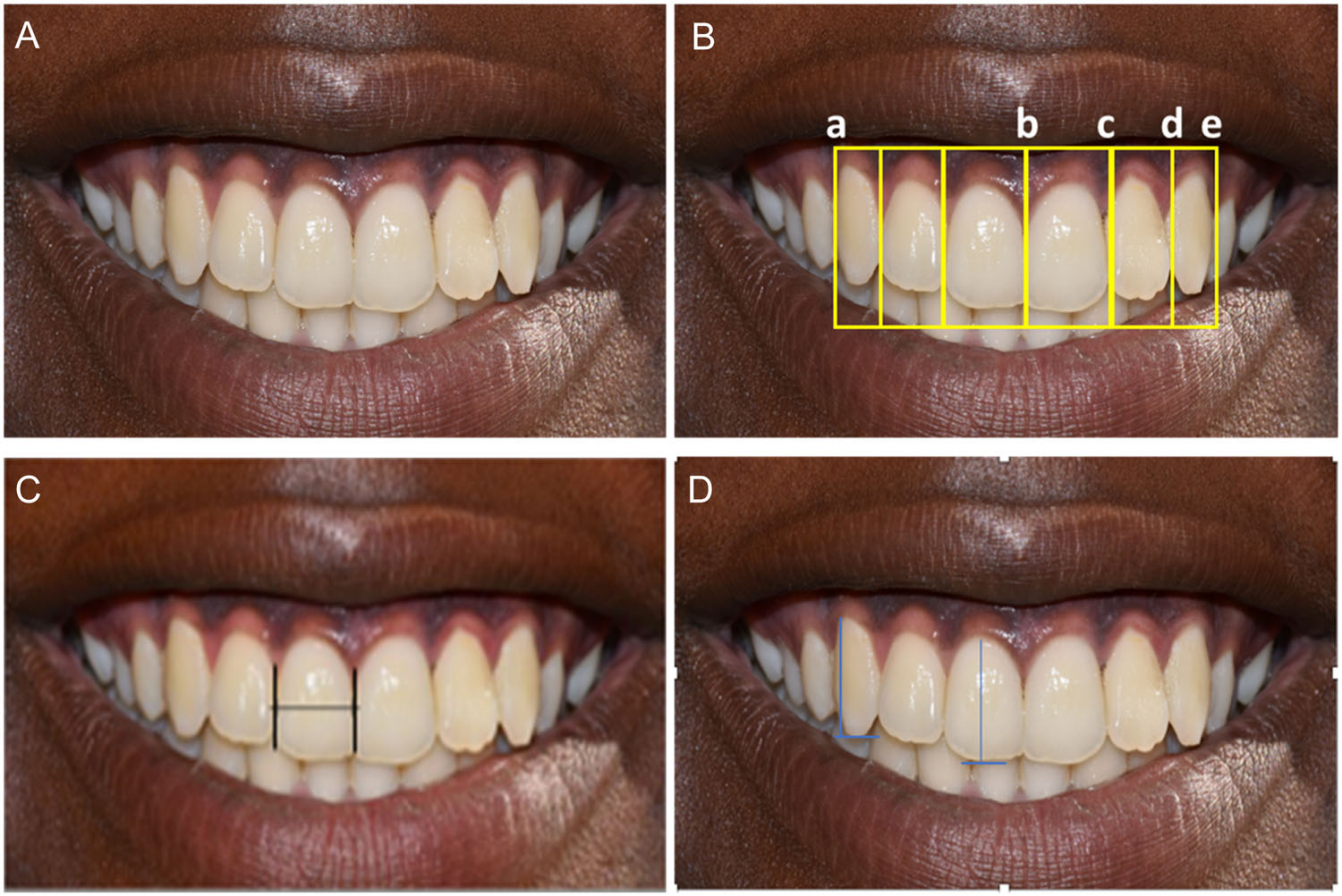
Illustration of the measuring points—A: widest smile displaying the maxillary anterior teeth; B—the widths of the maxillary incisors were measured at the mesio‐distal contact points of the teeth (b–c for central incisors and c–d for lateral incisors), the width of the canine was measured from the mesial contact point to the most distal point visible from the frontal view (d–e) while the frontal inter‐canine width was taken from the distal‐most visible point on the right canine to the left one (a–e); C—demarcation and measurement points of the width of 11; D—the heights of the teeth were measured from the gingival zenith to the incisal edge of the incisors or cusp tip of the canine.

The participants were seated on an ordinary chair with their heads in a natural head position (NHP) such that they looked forward. The NHP is a standardized and reproducible position of the head in an upright posture, with the eyes focused on a point in the distance at eye level (Jacobson 1995). Participants were asked to focus at a point on the wall located at eye level. A standardized frontal image of each participant's smile was captured using a calibrated digital camera, NIKON D3400 macro lens AF‐S VR MICRO NIKKOR 105 mm with a manual setting. To obtain a sharp facial image, the camera was adjusted to fit from the tip of the nose to the tip of the chin. The distance between the camera and subject was fixed at a working distance of 60 cm, while the camera was set to a focal length of 22 and a shutter speed of 1/100. Participants were asked to display their widest smile, as illustrated in Figure 2. Photographs were captured during the smile, and three images were taken per participant.

### 2.7 Procedure for Maxillary Anterior Teeth Measurements

The images were transferred to a computer, and measurements were performed using Adobe Photoshop 7 software (Adobe Studios, San Jose, California, USA). A digital Vernier caliper was used to measure the actual width of the sample teeth of one of the participants and was compared against the measurements obtained from Adobe Photoshop on the corresponding photographs, obtaining a conversion ratio of 0.1357. Standardization of the photographs and measurements was conducted by the first two authors M.N.M. and S.W.M. Subsequently, Adobe Photoshop was used to take three measurements of the described tooth parameters from all participant photographs, as illustrated in Figure 2, which were recorded in millimeters, and their means were calculated. The width of the maxillary incisor was measured at the mesiodistal contact points of the tooth, the width of the canine was measured from the mesial contact point to the most distal point visible from the frontal view, and the frontal inter‐canine width was measured from the most distal visible point on the right canine to the left one. The height of the teeth was measured from the gingival zenith to the incisal tip of the incisor or cuspal tip of the canine. These measurements were then used to calculate the Golden Proportion, RED Proportion, and Golden Percentage. Cohen's κ was used to determine inter‐rater reliability.

### 2.8 The Golden Proportion

To determine the Golden Proportion, the width of the maxillary lateral incisor was multiplied by 1.62 (Aziz and Hossain 2017; Preston 1993) and compared with the width of the adjacent central incisor. Further, the width of the maxillary canine was multiplied by 1.62 and compared with the width of the adjacent lateral incisor. Similar values indicated that the width of the canine was in Golden Proportion to that of the lateral incisor.

### 2.9 The Golden Percentage

The Golden Percentage was calculated by dividing the width of each maxillary central incisor, lateral incisor, and canine by the frontal inter‐canine width and multiplying the resulting value by 100 for each tooth. The obtained values were rounded off to the nearest whole number. If the values from canine to canine were 10%, 15%, 25%, 25%, 15%, and 10%, it indicated that the six maxillary anterior teeth were in the Golden Percentage.

### 2.10 The RED Proportion

The RED Proportion was calculated by dividing the width of each maxillary lateral incisor by the width of the adjacent central incisor and the resulting number multiplied by 100. Similarly, the width of each maxillary canine was divided by the width of the adjacent lateral incisor, and the resulting number was multiplied by 100. If the values obtained were constant and ranged between 60% and 80% (Ward 2001; Alhabahbah, Aburumman, and Al‐Shamout 2016; Sah, Zhang, and Chang 2014), it indicated that the central incisor, lateral incisor, and canine were in the RED Proportion.

### 2.11 The Width/Height Ratio

The width/height ratio was obtained by dividing the width by the height of each of the maxillary anterior teeth.

### 2.12 Data Analysis

Participants' data were serialized and Microsoft Excel 2013 (Microsoft Corporation, Redmond, Washington, USA) was used for data entry and cleaning. Data were coded and analyzed using the Statistical Package for Social Sciences (version 22, IBM Corp., Armonk, New York, USA) for Windows. Descriptive statistics included frequencies, proportions, means, standard deviations, medians, and interquartile ranges. To determine the validity of maxillary anterior teeth proportions, independent sample *t*‐tests were used to compare the means of tooth dimensions at *α* < 0.05.

## 3 Results

### 3.1 Sociodemographic Characteristics

A sample size of 175 participants was obtained. Of these, 107 (61.1%) were male with a mean age of 22.4 ± 3.5 years and 68 (38.9%) were female with a mean age of 22.4 ± 3.5 years. The majority of participants were aged between 18 and 25 years, with a mean age of 22.4 ± 3.5 years. The median age for all the participants was 22.0 (interquartile range 20.0–23.0) years.

### 3.2 Maxillary Anterior Teeth Widths/Heights

The inter‐rater reliability for the anterior tooth measurements was determined to be *κ* = 0.99, 95% CI: 0.83–0.99, *p* < 0.001. Descriptive summaries of the maxillary anterior tooth measurements are presented in Table 1. The mean widths of the anterior teeth on the right were 4.90 ± 0.76 mm for the canines, 5.90 ± 0.87 mm for the lateral incisors, and 8.60 ± 1.02 mm for the central incisors; while on the left, they were 4.83 ± 0.79 mm for the canines, 5.90 ± 0.88 mm for the lateral incisors, and 8.55 ± 1.03 mm for the central incisors. The mean tooth height‐to‐width ratios ranged between 0.55 ± 0.08 for the canines and 0.88 ± 0.09 mm for the central incisors.

**Table 1:** Descriptive summaries of maxillary anterior teeth measurements (mm) in 175 participants.

	Mean	SD	Median	IQR	Min	Max
*Tooth widths*						
Right canine	4.90	0.76	4.87	4.32–5.51	3.12	6.53
Right LI	5.90	0.87	5.97	5.24–6.53	3.92	8.09
Right CI	8.60	1.02	8.73	8.13–9.19	6.26	11.03
Left CI	8.55	1.03	8.64	8.04–9.21	6.16	11.13
Left LI	5.90	0.88	5.92	5.33–6.53	3.49	7.82
Left canine	4.83	0.79	4.87	4.27–5.43	3.22	6.79
ICD	38.70	4.63	39.24	36.76–41.82	28.04	49.82
*Tooth height/width ratio*						
Right canine	0.57	0.08	0.56	0.51–0.61	0.37	0.81
Right LI	0.73	0.09	0.73	0.68–0.79	0.60	0.96
Right CI	0.88	0.09	0.88	0.83–0.94	0.70	1.15
Left CI	0.87	0.09	0.86	0.82–0.92	0.68	1.15
Left LI	0.72	0.08	0.70	0.66–0.78	0.54	1.01
Left canine	0.55	0.08	0.54	0.50–0.60	0.36	0.77

Abbreviations: CI, central incisors; ICD, inter‐canine distance; IQR, interquartile range; LI, lateral incisors; SD, standard deviation.

The mean widths, heights, and inter‐canine distances of the maxillary anterior teeth according to sex are summarized in Table 2. There was no statistically significant difference in the mean tooth widths and inter‐canine distances which were similar in measurements for both sexes, although female participants had a wider inter‐canine distance than the male participants. However, for the mean tooth heights, there was a statistically significant difference between the left canines of the males and that of the females (*p* = 0.02).

**Table 2:** Mean widths, heights, and inter‐canine distances of maxillary anterior teeth by sex (mm) in 175 participants.

Mean widths and inter‐canine distances							
	Right canine	Right LI	Right CI	Left CI	Left LI	Left canine	Inter‐canine distance
Male	4.91 ± 0.77	5.91 ± 0.85	8.50 ± 1.03	8.46 ± 1.06	5.86 ± 0.91	4.84 ± 0.81	38.50 ± 4.78
Female	4.89 ± 0.76	5.88 ± 0.91	8.74 ± 0.98	8.69 ± 0.99	5.95 ± 0.83	4.82 ± 0.75	39.01 ± 4.40
*p* value	0.92	0.82	0.14	0.15	0.50	0.89	0.48

Mean heights						
	Right Canine	Right LI	Right CI	Left CI	Left LI	Left Canine
Male	8.88 ± 1.59	8.23 ± 1.28	9.91 ± 1.33	10.02 ± 1.36	8.39 ± 1.36	9.03 ± 1.55
Female	8.48 ± 1.23	7.94 ± 1.17	9.65 ± 1.35	9.69 ± 1.35	8.19 ± 1.22	8.54 ± 1.28
*p* value	0.06	0.08	0.22	0.12	0.34	0.02*

Abbreviations: CI, central incisors; LI, lateral incisors.

* *p* < 0.05.

### 3.3 Occurrence of the Golden Proportion, Golden Percentage, and RED Proportion

The Golden Proportion ratios of central to lateral incisors and lateral incisors to canines, RED Proportion values of central to lateral incisors and lateral incisors to canines, Golden Percentage values of central to lateral incisors and lateral incisors to canines, and the frequency of occurrence of these proportions are presented in Table 3.

**Table 3:** Mean Golden Proportion, RED Proportion, and Golden Percentage values and the occurrence for maxillary anterior teeth.

Mean Golden Proportion				
	CI to LI (Right)	LI to C (Right)	CI to LI (Left)	LI to C (Left)
Male	1.45 ± 0.13	1.22 ± 0.15	1.46 ± 0.14	1.22 ± 0.14
Female	1.50 ± 0.16	1.21 ± 0.16	1.47 ± 0.13	1.25 ± 0.15
Overall mean	1.47 ± 0.15	1.21 ± 0.15	1.46 ± 0.13	1.23 ± 0.15
*p* value	0.02*	0.86	0.47	0.26
*Occurrence in male participants*				
Present	1.1%	0.6%	1.7%	0.0%
Absent	98.9%	99.4%	98.3%	100.0%
*Occurrence in female participants*				
Present	2.9%	0.0%	1.1%	0.0%
Absent	97.1%	100.0%	98.9%	100.0%
*Occurrence in all participants*				
Present	4.0%	0.6%	2.8%	0.0%
Absent	96.0%	99.4%	97.2%	100.0%

Mean RED Proportion				
	LI to CI (Right)	C to LI (Right)	LI to CI (Left)	C to LI (Left)
Male	0.70 ± 0.06	0.84 ± 0.10	0.69 ± 0.06	0.83 ± 0.10
Female	0.67 ± 0.07	0.84 ± 0.11	0.68 ± 0.06	0.82 ± 0.10
Overall mean	0.69 ± 0.06	0.84 ± 0.11	0.69 ± 0.06	0.82 ± 0.10
Overall mean Proportion (%)	69.0 ± 6.0	84.0 ± 11.0	69 ± 6.0	82.0 ± 10.0
*p* value	0.02*	0.76	0.40	0.31
*Occurrence in male participants*				
Present	7 (4.0%)		2 (1.1%)	
Absent	168 (96.0%)		173 (98.9%)	
*Occurrence in female participants*				
Present	4 (2.3%)		1 (0.6%)	
Absent	171 (97.7)		174 (99.4%)	
*Occurrence in all participants*				
Present	11 (6.3%)		3 (1.7%)	
Absent	164 (93.7)		172 (98.3%)	

Mean Golden Percentage						
	Right C	Right LI	Right CI	Left CI	Left LI	Left C
Male	12.76 ± 1.38	15.35 ± 1.13	22.15 ± 1.16	22.00 ± 1.03	15.18 ± 1.12	12.58 ± 1.30
Female	12.59 ± 1.40	15.03 ± 1.37	22.43 ± 0.90	22.31 ± 0.89	15.26 ± 1.13	12.31 ± 1.37
Overall mean	12.69 ± 1.38	15.22 ± 1.23	22.26 ± 1.07	22.12 ± 0.98	15.21 ± 1.12	12.47 ± 1.33
*p* value	0.435	0.114	0.095	0.043*	0.618	0.190
Present	4 (2.3)	53 (30.3)	3 (1.7)	2 (1.1)	65 (37.1)	8 (4.6)
Absent	171 (97.7)	122 (69.7)	172 (98.3)	173 (98.9)	110 (62.9)	110 (62.9)

Abbreviations: C, canine; CI, central incisors; LI, lateral incisors.

* *p* < 0.05.

The overall mean Golden Proportion ratios of the central incisor to the lateral incisor, and the lateral incisor to the canine on the right were 1.47 ± 0.15 and 1.21 ± 0.15, respectively, while on the left, it was 1.46 ± 0.13 and 1.23 ± 0.15, respectively. There was a statistically significant difference in the mean Golden Proportion ratio of the central incisor to the lateral incisor on the right among males and females (*p* = 0.02). Nonetheless, the Golden Proportion was observed in only 1.1% in the central incisor to the lateral incisor, and 0.6% in the lateral incisor to the canine on the right, and in 1.7% in the central incisor to the lateral incisor on the left among male participants. Among female participants, the Golden Proportion was observed in only 2.9% in the central incisor to the lateral incisor on the right and 1.1% in the central incisor to the lateral incisor on the left.

The overall mean RED Proportion values of the lateral incisor to the central incisor, and the canine to the lateral incisor on the right were 0.69 ± 0.06 and 0.84 ± 0.11, respectively; while on the left, it was 0.69 ± 0.06 and 0.82 ± 0.10, respectively. There was a statistically significant difference in the mean RED Proportion of the lateral incisor to the central incisor on the right among males and females (*p* = 0.02). Nonetheless, the RED Proportion was observed in only 4% in the lateral incisor to the central incisor on the right, and 1.1% in the lateral incisor to the central incisor on the left among male participants. Among female participants, the RED Proportion was observed in only 2.3% in the lateral incisor to the central incisor on the right, and 1.7% in the lateral incisor to the central incisor on the left.

The overall mean Golden Percentage values of the canine, lateral incisor, and central incisor on the right were 12.69 ± 1.38, 15.22 ± 1.23, and 22.26 ± 1.07, respectively; while on the left, they were 12.47 ± 1.33, 15.21 ± 1.12, and 22.12 ± 0.98, respectively. There was a statistically significant difference in the mean Golden Percentage values of the left central incisor among males and females (*p* = 0.043). The Golden Percentage was observed in the right lateral incisor in 30.3% of the participants and in the left lateral incisor in 37.1% of the participants.

**Figure 3:**
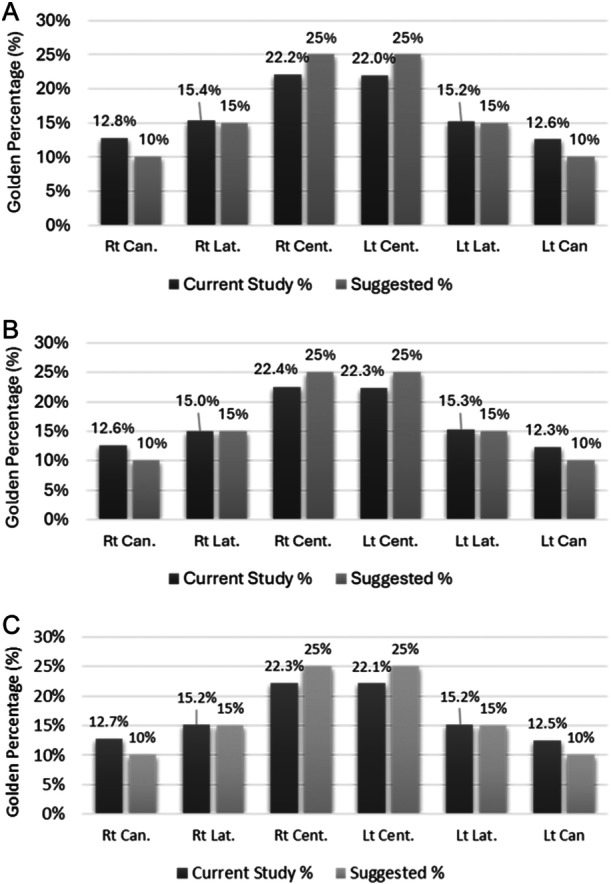
Comparison between the suggested Golden Percentage and that found in this study (A—male participants; B—female participants; C—all participants). Lt Cent, left central incisor; Lt Can, left canine; Lt Lat, left lateral incisor; Rt Can, right canine; Rt Cent, right central incisor; Rt Lat, right lateral incisor.

A comparison between the suggested Golden Percentage values and those obtained in this study is shown in Figure 3. As compared with the values proposed in the literature, the Golden Percentage values in this study were higher for the canines (12.31%–12.76%) and lower for the central incisors (22.0%–22.43%) but were in conformity for the lateral incisors (15.03%–15.35%).

### 3.4 Validity of the Golden Proportion, Golden Percentage, and RED Proportion Among 18–35‐Year‐Old Kenyans of African Descent

Table 4 shows the statistically significant differences between the tooth proportions of the population in the present study and the proposed Golden Proportion, Golden Percentage, and RED Proportion, indicating that they are invalid measurements within this population.

**Table 4:** Validity of the Golden Proportion, Golden Percentage, and RED Proportion.

Validity of the Golden Proportion				
Maxillary teeth	Mean	SD	*z*‐statistic	*p* value
CI to LI (right)	1.47	0.15	−13.40	<0.0001
LI to C (right)	1.21	0.15	−35.19	<0.0001
CI to LI (left)	1.46	0.13	−15.48	<0.0001
LI to C (left)	1.23	0.15	−35.48	<0.0001

Validity of the RED Proportion		
Maxillary teeth	*z*‐statistic	*p* value
Right	−16.15	<0.0001
Left	−15.47	<0.0001

Validity of the Golden Percentage				
Maxillary anterior teeth	Mean	SD	*z*‐statistic	*p* value
Canine (right)	12.69	1.38	25.72	<0.0001
Lateral Incisor (right)	15.22	1.23	2.39	0.02
Central Incisor (right)	22.26	1.07	−33.89	<0.0001
Central Incisor (left)	22.12	0.98	−38.72	<0.0001
Lateral Incisor (left)	15.21	1.12	2.49	0.01
Canine (left)	12.47	1.33	24.62	<0.0001

Abbreviations: C, canine; CI, central incisors; LI, lateral incisors; SD, standard deviation.

## 4 Discussion

This descriptive cross‐sectional study was the first to determine the Golden Proportion, Golden Percentage, and RED Proportion among Kenyans of African descent.

In the present study, the Golden Proportion between the maxillary central incisors and lateral incisors was found in 4.0% on the right and 2.8% on the left of all the participants, but between the maxillary lateral incisors and canines was found in only 0.6% on the right of male participants. A comparatively higher occurrence of the Golden Proportion has been reported among Asian and Arabic populations (Aziz and Hossain 2017; Ward 2001; Sulaiman et al. 2010; Alhabahbah, Aburumman, and Al‐Shamout 2016). For example, 16% of males and 18% of females have a Golden Proportion between the width of the central incisor and the width of the lateral incisor, and 2% of males and 6% of females have a Golden Proportion between the width of their lateral incisor and the width of their canine among the Bangladeshi population (Aziz and Hossain 2017). These different findings may be attributed to racial and ethnic diversities or differences in tooth form in different geographic regions. For instance, a set of dental proportion values for participants of African origin may not conform to the typical dentofacial features of participants of European, Asian, Arab, and other origins (Sah, Zhang, and Chang 2014). Although several authors have proposed the application of the Golden Proportion for achieving proportions and dental esthetics (Lombardi 1973; Levin 1978; Rosenstiel, Land, and Walter 2022; Rufenacht 1990; Shillingburg et al. 1997; Goldstein et al. 2018), the universal application of the Golden Proportion should be reconsidered because of the poor correlation between tooth dimensions, as found in various other studies (Mahshid et al. 2004; Preston 1993; Markovics, Jánosi, and Biriș 2013; Sulaiman et al. 2010; Sandeep et al. 2015; Fayyad, Jamani, and Aqrabawi 2006; Pesson et al. 2015; Ramani and Sreenivasan Murthy 2008) as well as in this study.

The present study reported that the RED Proportion between the maxillary lateral incisors and central incisors was in the range of 67.0%–70.0%, and the RED Proportion between the canine and lateral incisors was in the range of 82.0%–84.0%. In this study, the ratios between the central and lateral incisors, and between the lateral incisors and canines were not constant, because the ratio increased distally. Similar results have been reported in an Asian population (Ramani and Sreenivasan Murthy 2008). However, they found RED Proportions in the range of 69.5%–70.3% between the central and lateral incisors, which is slightly higher than the findings of the current study. The RED Proportion between the canine and lateral incisors was in the range of 80.0%–83.0%, which is slightly lower than the findings of the current study. Furthermore, others reported similar RED proportion values as those found in the present study, for the ratio between central and lateral incisors, and lower RED proportion values for the ratio of the width of the canine to the width of lateral incisors compared to those found in the present study (Aziz and Hossain 2017; Ward 2001; Fayyad, Jamani, and Aqrabawi 2006). These differences may be attributed to the non‐constant ratio between the central and lateral incisors and between the lateral incisors and canines. The values obtained in the present study suggest that the RED Proportion does not appear to exist in the natural dentition of Kenyans of African descent. This is because the ratios between the central and lateral incisors and those between the lateral incisor and canine were not constant. The results revealed that the ratio increases distally. Therefore, it can be concluded that the RED Proportion theory, as applied to natural dentition, may not be evident. This finding is also supported by several previous studies (Aziz and Hossain 2017; Fayyad, Jamani, and Aqrabawi 2006; Ramani and Sreenivasan Murthy 2008; Alhabahbah, Aburumman, and Al‐Shamout 2016; Sah, Zhang, and Chang 2014).

For the Golden Percentage, the results of the present study found that the mean value of the central incisors was 22.19%, which was lower than the suggested value of 25%. However, the mean value of the lateral incisors was 15.22%, which was like the recommended value of 15%. Additionally, the mean value of the canines was 12.58%, which was higher than the recommended value of 10%. The results of this study are similar to those reported in Asian (Ramani and Sreenivasan Murthy 2008) and Bangladeshi (Aziz and Hossain 2017) populations with 22.0% for central, 15.5% for laterals, and 12.5% for canines, and 22.67% for centrals, 15.36% for laterals, and 11.97% for canine, respectively. Furthermore, the results are similar to the findings in the Arabic population (Fayyad, Jamani, and Aqrabawi 2006) with 22.8%–23.0% for women's central incisors and 22.6% for men, and 14.6%–15.2% for women's lateral incisors and 14.59–15.09% for men, with only the canines being different (11.69%–11.89% for canines in females and 11.66%–11.87% for canines in males).

The similarity in the findings may be attributed to the existence of the Golden Percentage in the lateral incisors but not in the central incisors and canines. This variation in the percentages of central incisors and canines suggested by Snow in 1999 (Snow 1999) may be due to variations in the shape of the dental arch in the Kenyan population, resulting in a smaller percentage of central incisors and a larger percentage of canines. Therefore, it can be concluded that the Golden Percentage theory in relation to the lateral incisors was more applicable to the participants of this study. Based on the findings of the present and previous studies, there is evidence to support that the width of the central incisors is slightly smaller, while the width of the canines is slightly larger in the Kenyan population of African descent and the Indian and Bangladeshi populations (Aziz and Hossain 2017; Fayyad, Jamani, and Aqrabawi 2006; Ramani and Sreenivasan Murthy 2008). The values obtained in this study contradict the Golden Percentage theory suggested by Snow (Snow 1999). The percentages of central and lateral incisors and canines in the present study are consistent with those of previous studies (Aziz and Hossain 2017; Fayyad, Jamani, and Aqrabawi 2006; Ramani and Sreenivasan Murthy 2008). One study recommended a value of 23% for centrals, 15% for laterals, and 12% for canines (Fayyad, Jamani, and Aqrabawi 2006), while another recommended a value of 22% for centrals, 15.5% for laterals, and 12.5% for canines because these percentages are more applicable to natural dentition (Ramani and Sreenivasan Murthy 2008). Based on the findings of this study and related studies in various ethnic populations, we propose that a value of 22%, 15%, and 13% for the central incisors, lateral incisors, and canines, respectively, can be adopted.

It should be noted that the present study utilized data from Kenyan participants of African descent; thus, minor variations in the obtained values compared to previous studies (Snow 1999; Aziz and Hossain 2017; Fayyad, Jamani, and Aqrabawi 2006; Ramani and Sreenivasan Murthy 2008) may be attributed to the ethnic background of the participants. This might also explain the minor differences in previous studies’ conclusions regarding the presence or absence of the application of the Golden Percentage theory.

Finally, regarding the validity of these three proportions as determinants of dental esthetics among Kenyans of African descent, the results of hypothesis testing revealed a statistically significant difference between the recommended values of the Golden Proportion and those found in this study, indicating that the Golden Proportion did not exist among the study participants. This finding is in accordance with previous studies (Mahshid et al. 2004; Rufenacht 1990; Shillingburg et al. 1997; Preston 1993) where no reliable relationships with the average natural dentition were found. Similarly, when compared to the ideal RED Proportion theory, the findings of this study revealed a statistically significant difference between the maxillary anterior teeth on the right and left sides, with a lack of constant proportions. In addition, the RED Proportion was not constant in this study; the ratio increased as one moved distally. Therefore, based on the findings of this study and those of previous studies, there is no evidence to support the RED Proportion theory as applied to the natural dentition of the studied Asian, Indian, Arabic, Bangladeshi, and Kenyan populations (Aziz and Hossain 2017; Fayyad, Jamani, and Aqrabawi 2006; Ramani and Sreenivasan Murthy 2008; Pitti et al. 2011). Therefore, it was concluded that the Golden Proportion and RED Proportion were invalid determinants of maxillary anterior teeth proportions among Kenyans of African descent with naturally well‐aligned teeth. For the Golden Percentage, while the overall difference between the findings of all the teeth in this study and the recommended value was statistically significant, the values of the central incisors to the lateral incisors and the lateral incisors to the canines were close to the suggested value. In fact, the value of lateral incisors was the same as the ideal value on both the left and right sides. This finding is in accordance with previous studies (Aziz and Hossain 2017; Fayyad, Jamani, and Aqrabawi 2006; Ramani and Sreenivasan Murthy 2008) where a reliable relationship was found. Thus, it was concluded that the Golden Percentage is an invalid determinant of maxillary anterior teeth proportions among Kenyans of African descent with naturally well‐aligned teeth, except for the lateral incisors.

## 5 Conclusion

In the group investigated in this study, which may or may not have been representative of all Kenyans of African origin, it was found that a poor correlation exists between maxillary anterior tooth dimensions and the Golden Proportion, that the ratio between central and lateral incisors and between lateral incisors and canines is not constant and increases distally, and that the Golden Percentage exists only in lateral incisors but not the central incisors or canines among Kenyans of African descent with naturally well‐aligned teeth. It is recommended that these dental proportions be considered based on the racial and ethnic background of a population in different geographical regions to establish objectively quantifiable values helpful in esthetic restorations. Specifically, a Golden Percentage value of 22% for central incisors, 15% for lateral incisors, and 13% for canines has been recommended, as these percentages are more applicable to natural dentition and might serve as a guideline for creating harmonious proportions of maxillary anterior teeth in the Kenyan population of African descent.

## Author Contributions

Marion N. Mosomi, Susan W. Maina, Olivia A. Osiro, and Ben I. Omondi contributed to the conception and design, contributed to the acquisition, analysis, and interpretation of data, and drafted and critically revised the manuscript. All authors gave their final approval and agreed to be accountable for all aspects of the work.

## Conflicts of Interest

The authors declare no conflicts of interest.

## Data Availability

The data that support the findings of this study are available from the corresponding author upon reasonable request.
